# 

**Published:** 1874-02

**Authors:** 


### Books and Pamphlets Received.

History of the American Ambulance Established in Paris, during the Siege of 1870-71: together with the details of its Method and its Work. By Thomas W. Evans, M. D., D.D.S., Ph D., etc., London: 1873. New York: William Wood & Co. Buffalo: H. H. Otis.

The Nature of Gun-Shot Wonds of the Abdomen, and their Treatment. By Eugene Peugnet, M. D. New York: Wm. Wood & Co., 1874. Buffalo: H. H. Otis.

Clinical Researches in Electro-Surgery. By A. D. Rockwell, A. M., M. D., and Geo. M. Beard, A. M., M. D. New York: Wm. Wood & Co., 1873. Buffalo: H. H. Otis.

A Hand-Book of Medical and Surgical Reference. By John A. Wyeth, M. D. New York: Wm. Wood & Co., 1873. Buffalo: H. H. Otis.

A Clinical History of the Medical and Surgical Diseases of Women. By Robert Barnes, M. D., etc. Illustrated. Philadelphia: Henry C. Lea, 1874. Buffalo: Theo. Butler & Son.

The Students Guide to Surgical Anatomy. By Edward Bellamy, F. R. C. S., etc. Illustrated. Philadelphia. Henry C. Lea, 1874. Buffalo: Theo. Butler & Son.

A Practical Treatise on the Diseases of Children. By J. Forsyth Meigs, M. D., and William Pepper, M. D. Fifth Edition Revised and Enlarged. Philadelphia: Lindsay & Blakiston, 1874.

The Sphygmograph; Its Physiological and Pathological Indication. By Edgar E. Holden, A. M., M. D. Philadelphia: Lindsay & Blakiston, 1874.

Dictionary of Elevations and Climatic Register of the United States; containing in addition to Elevations, the Latitude, Mean Annual Temperature, etc. etc., with a brief Introduction. By J. M. Toner, M. D. New York: D. Van Nostrand, 1873.

THE BUFFALO

**Medical and Surgical Journal**

PUBLISHED MONTHLY,

Containing Original Articles, Reports of Medical Societies and Hospitals, Editorial, Reviews, Correspondence, Army News, etc., etc; including the usual variety of Medical Periodical Publications. Specimen copies sent on application.

Address Buffalo Medical & Surgical Journal, Buffalo, N. Y.

TERMS OF SUBSCRIPTION, - - $3.00 PER YEAR, IN ADVANCE.

